# Infant action understanding: the roles of active training and motor development

**DOI:** 10.3389/fdpys.2024.1349031

**Published:** 2024-05-13

**Authors:** Haerin Chung, Courtney A. Filippi, Amanda L. Woodward

**Affiliations:** 1 Laboratories of Cognitive Neuroscience, Division of Developmental Medicine, Boston Children’s Hospital, Boston, MA, United States; 2 Child Study Center, Department of Child and Adolescent Psychiatry, Grossman School of Medicine, New York University, New York, NY, United States; 3 Infant Learning and Development Laboratory, Department of Psychology, Division of Social Sciences, University of Chicago, Chicago, IL, United States

**Keywords:** action understanding, action perception, motor development, active training, actionperception link, infant

## Abstract

**Introduction:**

This study examined the potential interplay between motor development and intervention in support of action understanding.

**Methods:**

Eighty nine-month-old infants completed a tool-use training session and goal imitation paradigm that assessed action understanding in counterbalanced order. A metric of motor development was obtained using the Early Motor Questionnaire.

**Results:**

Results indicated that training improved action understanding, particularly for those infants who started out with lower means-end skills. Results further indicated that infants who did not receive any training experience in the lab beforehand, drew on their existing means-end skills.

**Discussion:**

These results emphasize independent contributions of training and motor development on action understanding and shed light on the interaction between training and individual motor readiness in facilitating action understanding in infancy.

## 1 Introduction

Every day, infants observe a wide range of actions—from simple goal-directed actions (e.g., reaching for a toy) to more complex actions where the goal is not the object being acted upon but using the object for another purpose (e.g., using a knife to slice an apple). To understand and respond to others’ actions, infants need to rapidly deploy knowledge about the goal structure of actions (Krogh-Jespersen and Woodward, 2014). This capacity is shaped by action experience (Hunnius and Bekkering, 2014; Woodward and Gerson, 2014; Gredebäck, 2018). Despite considerable interest in the link between action experience and action understanding, questions remain about how infants’ experiences across multiple timescales influence one another. The current study examines the interplay between infants’ starting state means-end skills and the effects of short-term action training on their action understanding.

The ability to discern the goal structure of others’ simple action (e.g., reaching) emerges early in life (Woodward, 1998), as revealed by converging findings across visual habituation, predictive looking, and imitation studies. By 5-to 6-months of age, infants show selective attention to the goal structure of action (Woodward, 1998; Hofer et al., 2007), generate anticipatory saccades to others’ action end-points (Cannon and Woodward, 2012; Ambrosini et al., 2013; Gredebäck and Falck-Ytter, 2015), and respond selectively by imitating others’ goals (Hamlin et al., 2008). For instance, Hamlin et al. (2008) showed 7-month-olds events in which an experimenter reached for one of two objects. When infants were given an opportunity to choose between the two objects, they systematically chose the experimenter’s prior goal. In contrast, when the experimenter did not act in a goal-directed manner (e.g., touching the toy with the back of her hand), infants chose randomly between the two objects. By 12-months of age, infants can discern the higher-order goals that structure sequences of actions (Sommerville and Woodward, 2005). When they observe someone using a cane to retrieve an out-of-reach toy, they understand that the person’s tool-use action is directed at the toy, rather than the cane itself. Infants under 12 months are limited in their ability to recognize the relation between tool-use actions on distant objects (Sommerville and Woodward, 2005; Cannon and Woodward, 2012; Gerson and Woodward, 2012). However, around their first birthday, sensitivity to goals extends to means-end action sequences that involves tool-use or intermediary objects (Hofer et al., 2005; Sommerville and Woodward, 2005; Henderson and Woodward, 2011).

An extensive behavioral literature has provided evidence for the link between action experience and action understanding in infants (Woodward, 2009; Hunnius and Bekkering, 2014; Gredebäck, 2018; Stapel, 2020). In this literature, “action experience” has been operationalized in two ways: (1) advances in infants’ motor development including grasping, means-end skills, or pointing (Sommerville and Woodward, 2005; Gredebäck and Kochukhova, 2010; Kanakogi and Itakura, 2011; Loucks and Sommerville, 2012; Melzer et al., 2012; Ambrosini et al., 2013; Filippi and Woodward, 2016), and (2) training interventions that introduce infants to new actions (Sommerville et al., 2005; Skerry et al., 2013; Gerson and Woodward, 2014a,b). In each case, findings have revealed connections between infants’ own skills or experience with an action that parallel their sensitivity to the goal structure of that action in others. To start, considerable evidence shows that as infants become more proficient in their own actions, they also become more adept in understanding the goal structure in others’ actions. For instance, Sommerville and Woodward (2005) measured infants’ spontaneous proficiency in means-end skills to retrieve a toy by pulling on the cloth on which it rested. They found that 12-month-olds as a group responded systematically to the means-end structure of others’ cloth-pulling action, but 10-month-olds did not. However, at 10 months, individual variation in infants’ own means-end cloth-pulling skills predicted their sensitivity to the goals of others’ cloth-pulling actions (Sommerville and Woodward, 2005). Other studies have revealed similar relations for pointing (Woodward and Guajardo, 2002; Brune and Woodward, 2007; Behne et al., 2012).

Other research has given infants brief training to provide them with the opportunity to learn novel actions that are within “reach” of their current motor skills. These intervention experiments show, for example, that infants who were trained on a means-end skill to use a cane to retrieve a toy (Sommerville et al., 2008), compared to those who passively observed an experimenter’s actions with the cane, became sensitive to the goal structure of others’ means-end cane-use actions. Similar effects have been observed for collaborative actions, in which two people play complementary roles to attain joint goal. Infants who first were given the opportunity to engage in the collaborative action subsequently showed greater sensitivity to collaborative goals in others’ actions (Henderson et al., 2013; Krogh-Jespersen et al., 2020).

In some training studies, infants who were more proficient in learning to produce means-ends actions during training also showed more sensitivity to others’ means-end goals (Sommerville et al., 2008). Relatedly, Gerson et al. (2015) showed a similar effect of learning to engage in a cloth-pulling action to retrieve an out-of-reach toy where infants’ variation in how well-infants learned during training predicted infants’ sensitivity to others’ means-end goals. Infants’ proficiency in means-end motor skills, alongside in-lab experiences with means-end tool-use, may influence their readiness to learn from training, which in turn, enhances the sensitivity to the goal structure in others’ actions. However, infant’s means-end skills prior to training was not evaluated in previous training studies, so it is difficult to disentangle this possibility.

Taken together, these findings indicate connections between infants’ own action experience (means-end skills and means-end training) and their goal understanding. Even so, they leave open questions about how these two aspects of experience—the effects of means-end skills and the effects of short-term means-end training—may relate to one another in supporting infants’ means-end goal understanding. That is, previous studies have not yet explored whether the extent to which infants benefit from training depends on the existing means-end skills they have accrued over development. This is an intriguing unanswered question as infants’ experience via advances in motor development and short-term training differ in many aspects. First, motor maturity unfolds over a prolonged developmental time scale, with emerging new skills (i.e., grasping, means-ends, pointing) becoming precise, refined, and consistent and incorporated into the infants’ motor repertoire over many months (Sommerville et al., 2005). Contrary to motor development, training in the relevant studies usually involves a single, brief exposure to the new action. These opportunities may provide infants motoric experience with an action that is within but not yet established in their motor repertoire, and provide an opportunity to link motoric experience to the effect of their motor action. Training could thus facilitate anticipation of the actor’s goals and this process could potentially be expedited during training intervention, where the experimenter provides assistance as needed, compared to the developmental time period it would typically take for infants to acquire these skills. Moreover, the two kinds of experiences also likely relate to different underlying neurocognitive processes. For instance, the prefrontal and motor areas play an important role during execution of stable and well-practiced skills, while the frontal and cerebellum is suggested to play a critical role during the early stages of learning a new skill (Doyon et al., 2002; Halsband and Lange, 2006; Nishiyori, 2016). Thus, it is yet an open question to explore the contributions of different types of experience on action understanding.

These points highlight the need for a closer look at infants’ motor development and training in one study, which could shed light on whether and how specific aspects of motor development and training interact to affect action understanding. Due to the challenges in assessing several infant capacities in a short time given infants’ limited attention spans, parent report questionnaires such as the Early Motor Questionnaire (Libertus and Landa, 2013) are fruitful to assess a broad range of skills, which may not be captured in a single lab session (encompassing gross motor, fine motor and visual receptive skills). We sought to capture infants’ means-end problem-solving skills via a set of questions on the visual-reception scale within the EMQ. Moreover, the breadth of EMQ allows to ask whether means-end skills in particular, or rather motor development in general, facilitates means-end action understanding.

### 1.1 Current study

The present study investigated the effects of means-end training and means-end skills on means-end goal understanding, within the same experiment. To do so, we assessed infants’ starting-state means-end problem solving skills with a validated parent report questionnaire (EMQ) prior to in-lab assessments, conducted an in-lab means-end training to teach infants to retrieve out-of-reach toys with a novel cane (modeled after Sommerville et al., 2008), and evaluated means-end goal understanding using a goal imitation paradigm (Hamlin et al., 2008).

We aimed to investigate whether there is an interaction between short-term means-end training and means-end skills on infants’ means-end goal understanding. One possibility is that there is an additive interaction between training and means-end skills, such that those infants who arrive at the lab more proficient in the relevant motor skills would benefit more from the training. In this case, motor advances in means-end skills might serve as an index of infant’s “readiness to learn” (Piaget, 1955; Lockman, 2000; Keen, 2011; Gerson et al., 2015), and may mediate learning during training and subsequently enhance goal understanding. Alternatively, there may be a compensatory interaction—infants who come in with less advanced means-end skills benefit more the from training in terms of its effects on goal understanding. A final possibility is that the effects of motor skill and training could be independent, such that training improves infants’ goal understanding independent of their starting skill level.

Along with our main questions, we expected to replicate prior findings illustrating that (1) training alters infants’ goal understanding of novel actions (2) the more proficient an infant is at producing the trained action, the more adept they are in recognizing this action as goal-directed (Sommerville et al., 2008; Gerson et al., 2015), and (3) infants’ own competence in a specific action is associated with recognizing that action as goal-directed (Kanakogi and Itakura, 2011; Cannon and Woodward, 2012; Melzer et al., 2012). In addition, we had two exploratory questions regarding infants’ motor development. First, as variations in motor development affects learning and cognition (Adolph and Joh, 2007) we explored whether means-end skills measured via the EMQ predicted infants’ degree of learning during means-end training. Second, no study has asked whether means-end skills specifically correlate with means-end goal understanding, as aforementioned studies have only examined variability in infants’ own actions in a single domain (often using the exact task that children will see during the action observation assessment). Therefore, we explored whether means-end skill is a specific predictor of means-end goal understanding or motor maturity in general contributes to means-end understanding, using the subscales within the EMQ.

## 2 Method

### 2.1 Participants

Eighty full-term (minimum 37 weeks gestation) 9-month-old infants (Mage = 8 months 27 days; range = 8 months 0 days−10 months 0 days, 39 female) participated in this study. Participants were recruited from a database of families in a large Midwestern city in USA interested in participating in child development research, and represented a diverse racial background (44% White, 13% African American, 6% Asian, 5% Hispanic, and 33% more than one race). Parent/guardian consent was obtained for all infants and were from highly educated backgrounds (maternal education: 77% with a bachelor’s degree or higher education). Forty infants were randomly assigned to the training-first condition (20 females; *M* = 8 months 24 days) and 40 to the imitation-first condition (19 females; *M* = 8 months 27 days). An additional 23 infants were tested but not included in final analyses due to failure to complete either imitation or training session (*N* = 10), inattention during the task (*N* = 3), side bias (choosing the same side across all six trials) during imitation procedure (*N* = 7), technical issues (*N* = 2), or parents not consenting to session being filmed (*N* = 1). The conditions did not vary in terms of average age or sex composition (all *ps* > 0.8). Data loss, age, and sex of the infant did not differ as a function of condition (all *ps* > 0.8).

The expected sample size necessary to detect a moderate effect size of 0.25 with significance level of *α* = 0.05 and a power of 0.8 in a multiple regression with two predictors is calculated to be 42. Our current sample size exceeds this requirement by nearly double, potentially allowing for increased statistical power to detect the effects of interest.

### 2.2 Procedure

Infants participated in a means-end tool-training paradigm (i.e., cane training task) and a goal imitation task (used to evaluate goal understanding) in one lab visit. Infants were either randomly assigned to undergo tool training before (training-first condition) or after (imitation-first condition) the goal imitation procedure. Including these two conditions allowed us to (1) evaluate the effect of training, and (2) evaluate whether the EMQ scores predict goal understanding, prior to any training with the imitation-first condition. For both tasks, infants sat on a parent’s lap at a table and parents were asked not to influence their infant’s actions in any way.

#### 2.2.1 Cane training task

The cane training task was modeled after Sommerville et al. (2008). During the task, infants pulled a cane to retrieve a series of out-of-reach toys. All infants were given pre-training and post-training trials in which the experimenter did not help the infant in any way. The pre-training trials indexed infant’s knowledge of how to use the cane prior to the intervention and the post-training trials indexed learning over the training session. Between pre-training and post-training, the experimenter provided infant-directed training that supported the infants’ success in retrieving the out-of-reach toy themselves (using the cane). During all three phases of the training task, the experimenter gave the toy back to the infant after each trial was over, even if the infant was not able to retrieve the toy independently, to keep infants motivated. Infants acted on a red cane during pre-training and training, and a blue cane (which was identical to the red in every way except for color) for post-training trials (the blue cane was used as a test of how well the infant was able to generalize what they learned when working with the red cane to a new cane).

##### 2.2.1.1 Experimental set-up

**FIGURE 1:**
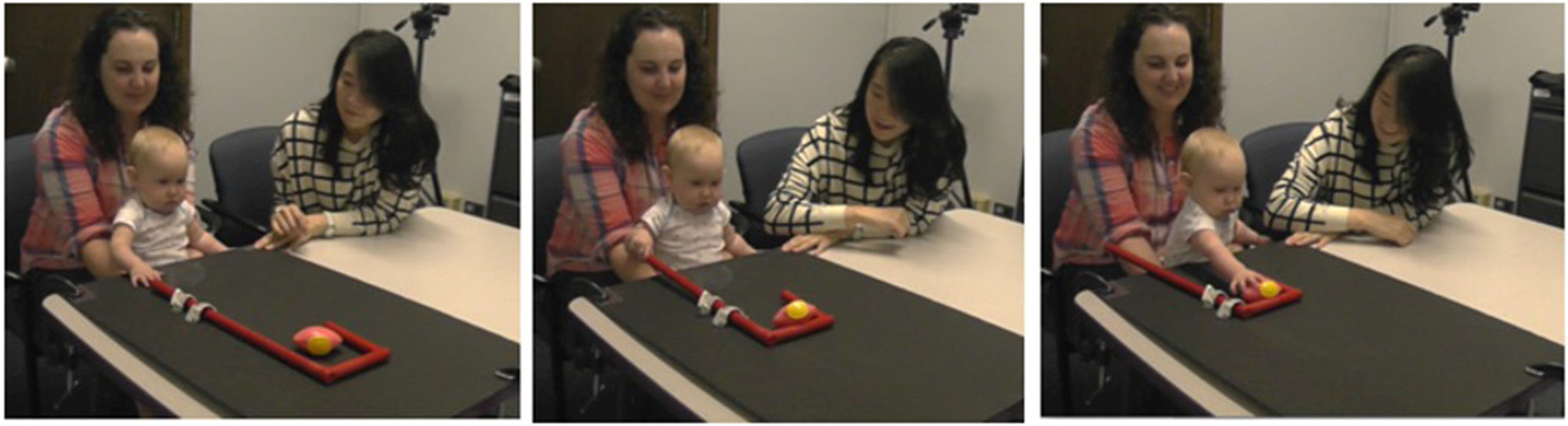
Picture of the cane training task.

Infants were seated within reach of the cane with enough room to pull the cane (length of cane: 48 cm, width of crook: 13.5 cm). The experimenter sat beside the infant so that the infant and the experimenter were both facing the toy and the cane (see Figure 1). A camera was placed in front of both the experimenter and infant to record the training session. Recordings were then used for offline coding.

##### 2.2.1.2 Pre- and post-training trials

Infants received three pre-training and three post-training trials in which the experimenter placed the toy (animal shaped bath toys) out of the infants’ reach in the crook of the cane. Infants acted on a different toy on each trial, and toys were presented in random order. Each trial began with the experimenter placing a toy in the crook of the cane. Pre- and post-training trials ended after infants either successfully retrieved the toy or 30 s elapsed indicated by a stopwatch. If an infant was successful at retrieving the toy, the experimenter provided positive encouragement (i.e., “Good job” or “Yay”). If an infant was unsuccessful, the experimenter provided a positive comment and gave the toy to the infant (i.e., “Let’s try this one.”). If an infant was successful at using the cane to retrieve the out-of-reach toy on all three pre-training trials, the experimenter proceeded directly to post-training (skipping training trials; training-first: *N* = 4; imitation-first: *N* = 8).

##### 2.2.1.3 Training trials

Immediately following pre-training, infants were taught how to retrieve toys using the cane. During training, the experimenter helped the infants to pull the cane to retrieve the toy themselves. Types of assistance included tapping on the toy, tapping on the cane, helping infants to pull the cane, modeling cane-pulling, and praising infants after they obtained the toy. Assistance was adjusted depending on the infants’ behaviors (e.g., if the infant did not reach for the cane, the experimenter tapped on the cane’s handle). Each training trial ended after infants either successfully retrieved the toy or 30 s elapsed. Infants proceeded to post-training if they either (1) used the cane to retrieve the out-of-reach toy on at least three trials (this included successful trials during the pre-training and the three trials need not be consecutive) or (2) reached a maximum of eight training trials.

#### 2.2.2 Goal imitation task

A modification of the goal imitation task developed by Hamlin et al. (2008) was used as a measure of infant’s goal understanding. This task has been utilized at several ages to evaluate infants’ goal understanding (Hamlin et al., 2008; Mahajan and Woodward, 2009; Gerson and Woodward, 2012).

##### 2.2.2.1 Experimental set-up

Infants sat on their parent’s lap in front of a table and the experimenter sat across from the infant facing them. Two cameras were used to record this task; one camera was placed behind the experimenter to record the infant’s behavior and another camera was placed behind the infant to capture the experimenter’s demonstration. These recordings were used for offline coding.

##### 2.2.2.2 Familiarization phase

Infants were first familiarized with the twelve bath toys (which differed from the toys used in cane training task) that would be featured during the goal imitation procedure. Each toy was presented one at a time in randomized order, on alternating sides of a board (76 × 23 cm). Trials ended when infants manually touched the toys. If the infant did not touch the toy, the experimenter handed the toy to the infant so the infant knew they could pick up the toys.

##### 2.2.2.3 Test phase

**FIGURE 2:**
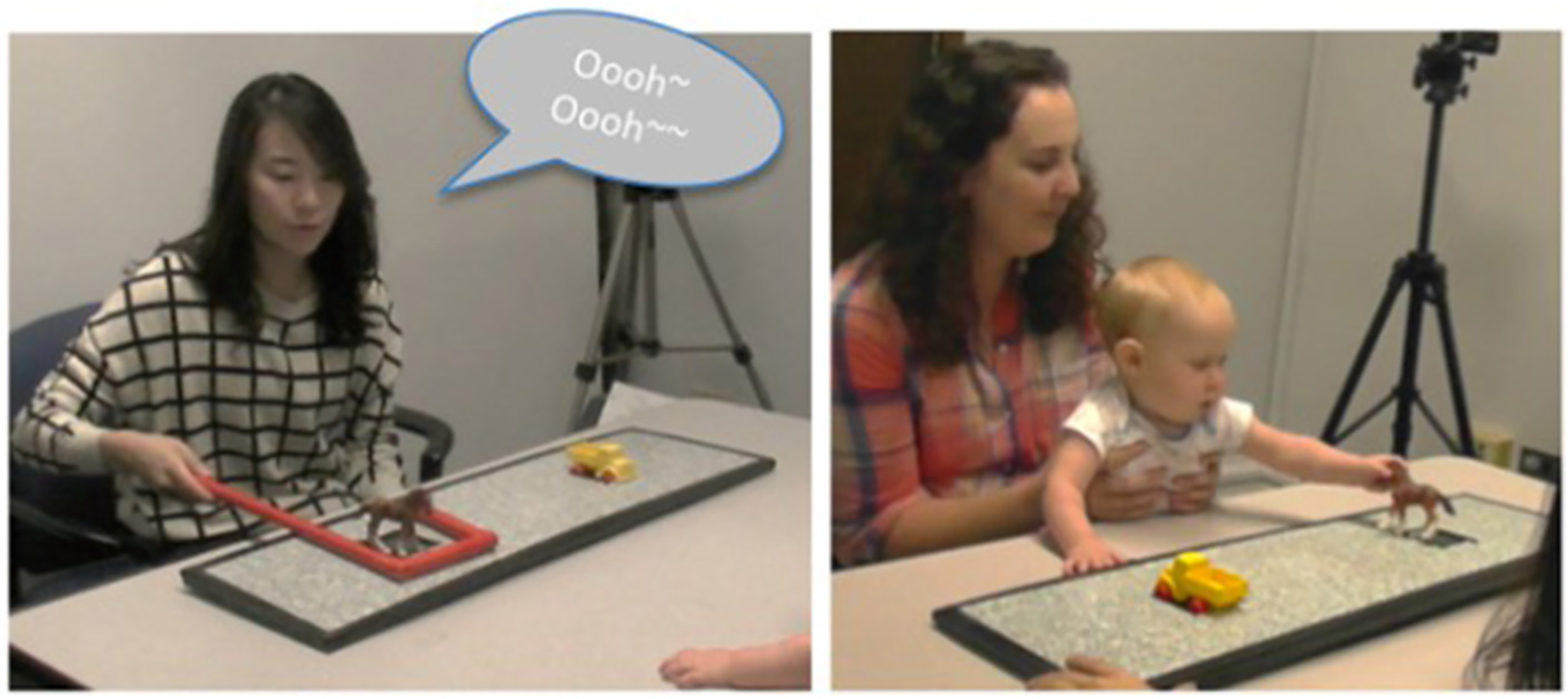
Picture of the goal imitation paradigm. **(Left)** Screenshot of experimenter using the cane to indicate the goal toy. **(Right)** Screenshot of an infants’ subsequent goal response.

Following familiarization, the experimenter placed a pair of toys 28 cm apart on the tray (see Figure 2) behind an occluder. Once set up, the experimenter removed the occluder and monitored the infants’ gaze to ensure the infant looked toward both toys. If infants did not look toward to the toys, the experimenter snapped or clapped behind each toy to direct the infant’s gaze to it. She then brought the infants’ gaze to the center by saying, “Hi! Look!” She then shifted her gaze toward the goal toy as she placed the crook of the red cane (i.e., the cane used during training trials) around one of the two toys to indicate her toy choice. She gazed at the toy throughout and said “Oooh!” twice but did not pick up or move the toy. The experimenter then withdrew the cane and established eye contact with the infant, bringing the infant’s attention back to center. She then looked down at the table (to ensure her gaze could not influence the infant’s subsequent choice), pushed the tray to the infant, and said, “Now it’s your turn!” The experimenter ended the trial when the infant selected a toy or, if the infant did not act on either toy, after 30 s elapsed. This procedure was repeated six times with a new pair of toys presented at each trial. The order of the pairs was pseudo-randomized. Within each condition, each toy was selected by the experimenter equally, and side of presentation and side of first reach was counterbalanced across infants.

#### 2.2.3 Early Motor Questionnaire

To assess infants’ motor development, we administered the Early Motor Questionnaire (EMQ) developed by Libertus and Landa (2013). The EMQ is a parent report measure that has been validated using the Mullen Scales of Early Learning (Mullen, 1995) and Peabody Developmental Motor Scales (Folio and Fewell, 2000). The items included on the EMQ describe motor behaviors typically emerging within the first 2 years of life (0–24 months). Parents report their infant’s gross motor (GM), fine motor (FM), and visual receptive (VR; formally known as perception-action scale). The EMQ uses a 5-point scale ranging from −2 to +2. The score−2 indicates if the parent is sure the infant does not show the behavior yet, and +2 if parent remembers a particular instance where the infant exhibited the behavior. Parents filled out the EMQ online prior to their visit to the lab and if parents were unable to complete the EMQ prior to the visit, they filled it out in the waiting room during consent prior to the experiment.

### 2.3 Coding

#### 2.3.1 Cane training task

##### 2.3.1.1 Planful toy retrieval

To assess learning over the course of the training session, we coded for the proportion of trials that the infant used the cane to retrieve the out-of-reach toy in a goal-directed or planful manner during pre-training and post-training. Trials were scored as planful if infants looked at the toy, pulled the cane while maintaining attention to the toy, and grasped the toy within 3 s (see Sommerville and Woodward, 2005; Sommerville et al., 2008; Gerson and Woodward, 2012 for comparable coding scheme). Reliability coding was completed for the whole sample for the pre- and post-training trials with two independent coders. A moderate-to-high degree of reliability was found for both planful scores in pre-training (ICC- 0.79, with 95% confidence interval from 0.69 to 0.84) and post-training (ICC- 0.78, with 95% confidence interval from 0.67 to 0.85) trials.

##### 2.3.1.2 Experimenter assistance

To evaluate the extent to which the experimenter assisted the infant during training, we coded the training trials to identify the frequency of each method of training support that the experimenter could provide. Coders identified instances of: tapping on the toy, assisting the infant in pulling the cane, and changing the cane’s angle. These data were used to determine whether any of these experimenter variables accounted for infants’ improvement from pre- to post-training trials.

#### 2.3.2 Goal imitation task

##### 2.3.2.1 Goal imitation

In this procedure, goal imitation, an indicator of goal understanding, refers to choosing the same goal object as the experimenter (Hamlin et al., 2008; Gerson and Woodward, 2012). Coders, who were unaware of infants’ condition or the experimenter’s goal on each trial coded infants’ toy selection offline using video. The infant’s choice was coded as the first toy touched so long as the infant visually attended to the toy before the touch (Gerson and Woodward, 2012). If the infant touched a toy without looking or touched the toy by accident, this was coded as a mistrial. If the infant did not choose a toy, the trial was coded as no choice. All mistrials and no choice trials were excluded from subsequent analyses. A second independent coder scored 25% of the infants, and the two coders’ judgments were highly correlated [Cronbach’s alpha (a) = 0.97].

##### 2.3.2.2 Infant attention

Infants’ visual attention during the goal imitation task was also coded to assess whether attention differed as a function of condition. Coders identified the duration of the time that the infant attended to the goal toy, non-goal toy, and experimenter using a digital coding program (Mangold, 2022). A second independent coder coded 25% of the infants’ test-trials and the two coders’ judgments were highly correlated (Attention to goal toy: ICC- 0.94 with 95% confidence interval from 0.82 to 0.98; experimenter: ICC- 0.86 with 95% confidence interval from 0.6 to 0.96; other toy: ICC- 0.90 with 95% confidence interval from 0.72 to 0.97). The relative time to aspects of the events (goal toy, non-goal toy, and experimenter) were calculated by each aspect of the event divided by the total duration of attention.

## 3 Results

All statistical analyses (packages: ggstatsplot, stats package) and visualizations (packages: ggplot2, cowplot, ggstatsplot) were done using R programming language (R Core Team, 2021).

### 3.1 Preliminary analyses

Preliminary analyses were conducted to evaluate the relation between all measures of interest (goal imitation, planful scores, and EMQ scores), and, respectively, age and sex. Results indicated that infants’ goal responses (during the goal imitation task) nor pre-test planful scores were not related to age [*F*_(1, 77)_ = 0.14, *p* = 0.705; *F*_(1, 74)_ = 0.13, *p* = 0.714, respectively] or sex (Kruskal-Wallis chi-squared = 2.6, *p* = 0.107; Kruskal-Wallis chi-squared = 0.99, *p* = 0.315, respectively). Results indicated that infants’ post-test planful scores were not related to age [*F*_(1, 70)_ = 1.59, *p* = 0.212] but related to sex (Kruskal-Wallis chi-squared = 4.86, *p* = 0.027). Importantly, there were equal numbers of females and males in each condition. When we conducted a linear regression with sex and condition as independent variables and Post-test scores as dependent variable, we no longer saw the effect of sex (*β*= −1.67, *p* = 0.316). EMQ scores did not differ as a function of sex (all *p*s > 0.5), though scores were related to age (GM: *r* = 0.4, *p* < 0.001; FM: *r* = 0.25, *p* = 0.033; VR: *r* = 0.23, *p* = 0.043). While we did not see differences between age [*t*_(72)_ = −0.23, *p* = 0.815] nor EMQ scores (all *p*s > 0.3) between conditions, we included age as covariate in our focal analysis.

#### 3.1.1 Goal imitation

Preliminary analyses indicated that infants’ responses did not vary across trials [*F*_(5, 57)_ = 0.99, *p* = 0.42] thus average goal imitation across all six test trials was used in all analyses. There were no condition differences in the number of mistrials (*p* > 0.831) or no choice trials (all *p*s > 0.599).

#### 3.1.2 Learning during cane training task

The number of training trials infants received did not differ between conditions (training-first condition: *M* = 2.2; range: 0–7 trials; imitation-first condition: *M* = 2.0; range: 0–7 trials). On average, the training session lasted 3 min and 32 s (range: 30 s to 6 min and 40 s). The level of experimenter’s assistance did not differ between condition (all *p*’s > 0.2). We next tested whether infants learned over the course of the cane training session by comparing infants’ planful scores at pre-training to post-training. Paired Wilcoxon signed-rank test indicated that infants improved from pre-training to post-training in their ability to retrieve the toy using the cane in the training-first condition but not in imitation-first condition (training-first condition: pre-training (*M* = 0.48, *Median* = 0.33, *SD* = 0.35), post-training (*M* = 0.65, *Median* = 0.67, *SD* = 0.36); *V*_*Wilcoxon*_ = 99.5, *p* < 0.001; imitation-first condition: pre-training (*M* = 0.51, *Median* = 0.67, *SD* = 0.39), post-training (*M* = 0.52, *Median* = 0.67, *SD* = 0.42); *V*_*Wilcoxon*_ = 81, *p* = 0.38). This unexpected result demonstrates that the effect of training was greater for those in the training-first condition. We evaluated whether this could be due to differences in experimenters’ assistance—it was not. We additionally verified that the amount of experimenter’s assistance was associated with post-test planful score. Associations between experimenter assistance during training and post-test scores did not differ by condition (see Supplementary material for details). Therefore, we suspect that these differences are likely related to fatigue following the imitation paradigm (i.e., for the imitation first condition). Importantly, the lack of evidence of learning in the imitation-first condition did not create a problem because the aim of our study was to directly test the effect of receiving training prior to the imitation task vs. not.

#### 3.1.3 EMQ

For our main analysis, we took a subset of the VR scale that could be indicative of infants’ means-end problem solving skills. These were the items that were selected because they reflect individual variation in infants’ means-end motor skills relevant to the cane training task and goal imitation task (referred to as ”EMQ-Means End score” from now on; #7~13 within the VR sub-scale; e.g., pull on a string or cloth to obtain a connected object). In order to check whether EMQ-Means End scores differed between conditions, we conducted Wilcoxon rank sum test. The median of EMQ-Means End score did not differ between conditions (Training-first: *Median* = 11.03, *SD* = 2.25; Imitation-first: *Median* = 11.2, *SD* = 2.55). See Table 1 for a summary of raw EMQ scores and Table 2 on for the details on the selected items for the EMQ-Means End score within the VR scale.

#### 3.1.4 EMQ-Means End score as a unique predictor

With the aim to verify that the EMQ-Means End items we have selected to reflect means-end problem-solving skills are unique predictors over and other “motor maturity” items in the EMQ, we conducted a stepwise regression (backward selection) starting with all the EMQ subscales (GM, FM, visual reception items not included in the “means-end” sub-subscale (Non_MeansEnd), and Means End scores) entered in the model with condition as predictors and goal imitation score as the dependent variable (See Supplementary material 3. Stepwise regression conducted with all the EMQ subscales). The analyses provided evidence of the unique contribution of the EMQ means-end items and their relations to the goal understanding and training. Thus, for our focal analyses, we focus on our EMQ means-end scores as a predictor of goal understanding.

### 3.2 Focal analyses: interplay between means-end skills and training on goal imitation

To explore the interplay between infants’ means-end training and means-end skills on goal understanding. We conducted a multiple regression analysis with condition (training-first, imitation-first) and EMQ-Means End scores (individual variation in infants’ means-end motor skills) as factors in predicting goal understanding (proportion of goal imitation), and age as covariate.

**FIGURE 3:**
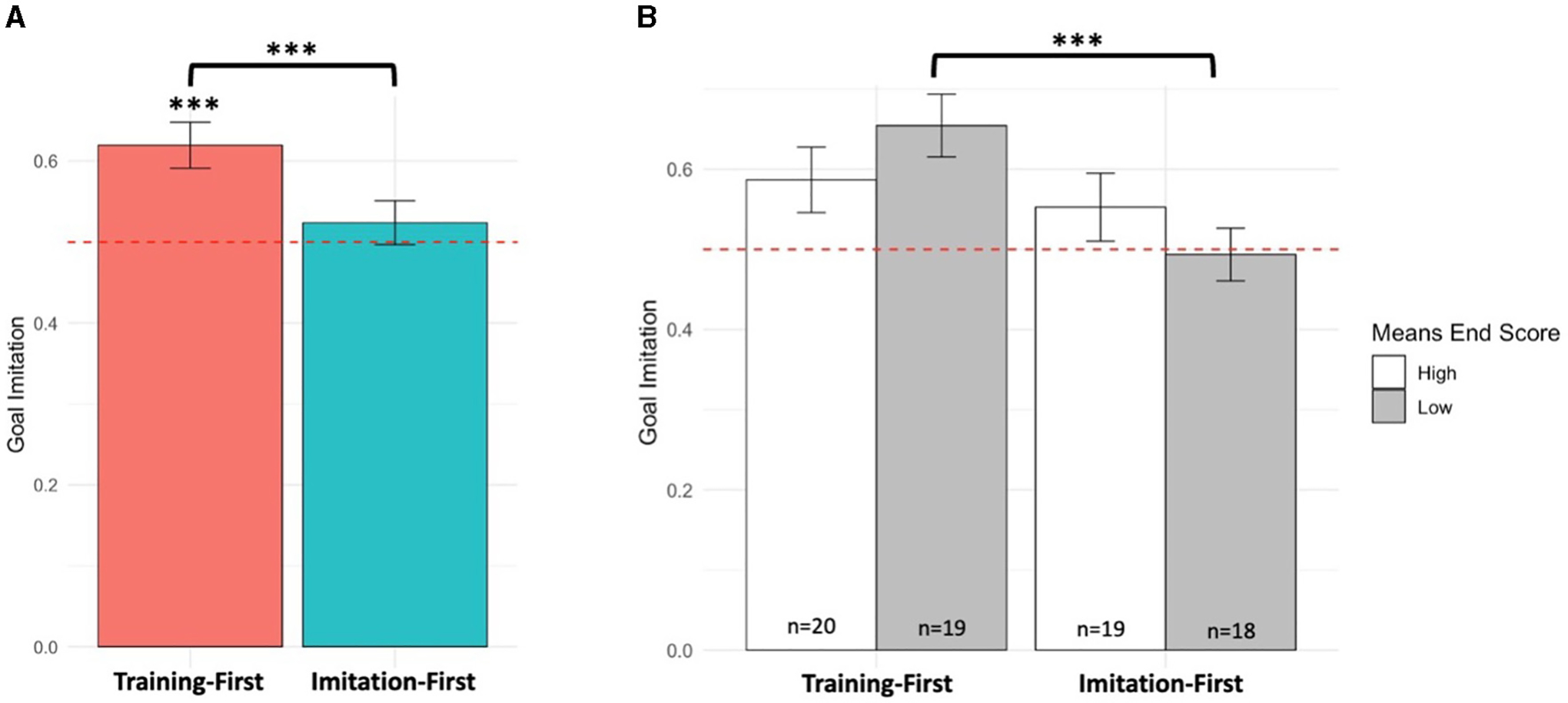
Proportion of infants’ goal imitation by **(A)** conditions and **(B)** EMQ-Means Ends scores (median split). Red dashed horizontal line indicates the chance level of 50%. Error bars indicate ± 1 standard error. ***p < 0.001.

The model reached significance [*R*^2^ = 0.16, *F*_(4, 71)_ = 3.38, *p* = 0.014], indicating a significant main effect of condition (ß = −0.54, *SE* = 0.17, *p* = 0.002), a significant main effect of Means-end scores scores (ß = −0.61, *SE* = 0.26, *p* = 0.022), and a significant interaction of condition and EMQ-Means End scores (ß = 0.40, *SE* = 0.15, *p* = 0.010). A closer look at the main effect of condition revealed that infants in the training-first (*M* = 0.63, *SD* = 0.18) condition chose the goal significantly more often than infants in imitation-first (*M* = 0.52, *SD* = 0.16) condition [mean difference = 0.11; *t*_(78)_ = 2.92, *p* < 0.001; *d* = 0.48; additionally confirmed with non-parametric test *W*_*Mann*−*Whitney*_ = 3,774, *p* < 0.001; Figure 3A]. Furthermore, infants in the training-first condition imitated the goal more often than would be expected by chance [one-sample *t*-test against chance level: *t*_(39)_ = 4.56, *p* < 0.001]. This was not found in the imitation-first condition [*t*_(39)_ = 0.61, *p* = 0.545].

**FIGURE 4:**
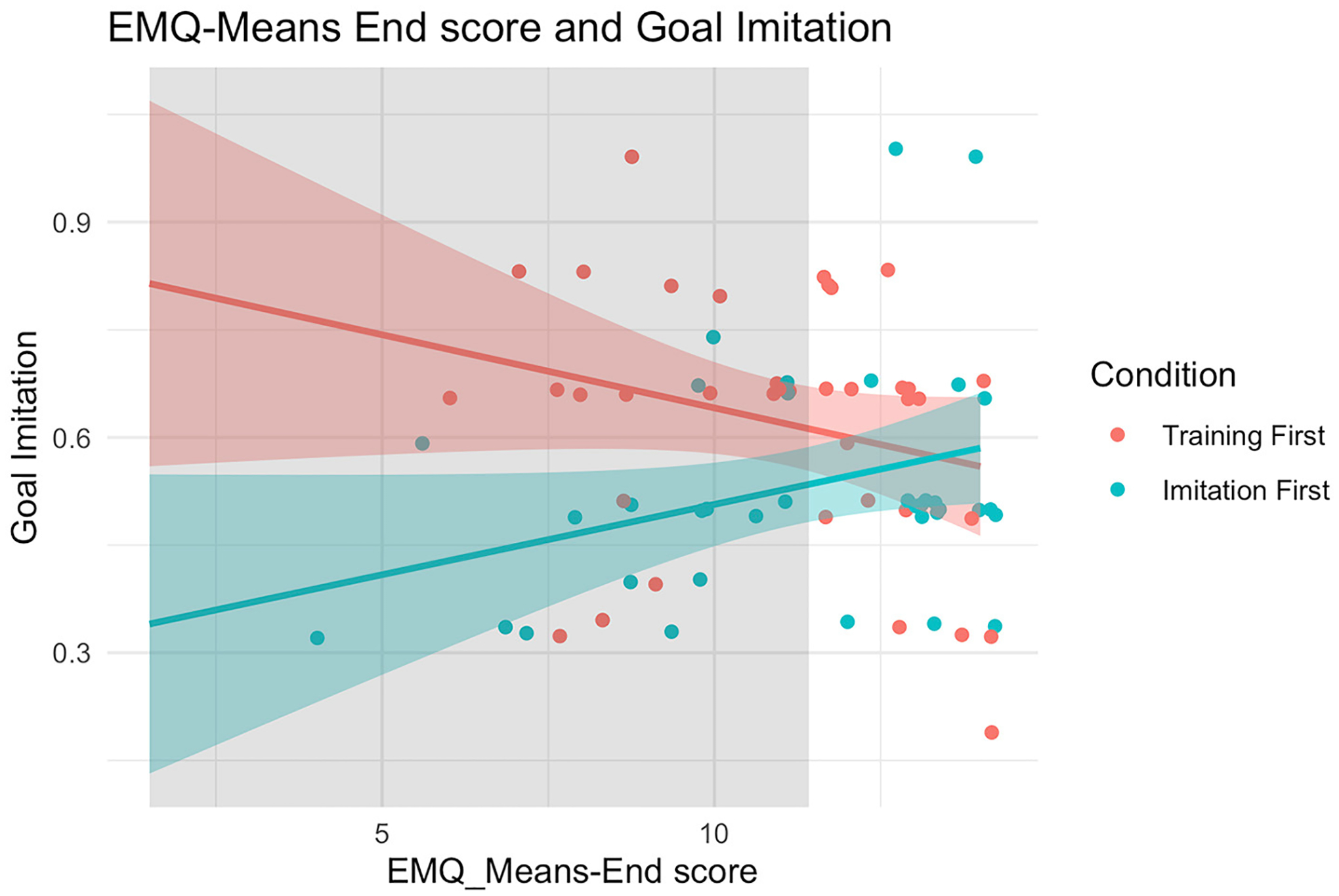
Scatterplot of infants’ goal imitation and EMQ-Means End scores separated by condition. Region of significance is depicted in the light gray range (EMQ-Means End score under 11.03).

Two follow-up analyses were performed to determine where the interaction effect originated. First, the Johnson-Neyman technique (Johnson and Neyman, 1936) was adopted to further establish the region of significance of the interaction, by identifying the values of EMQ-Means End scores that are associated with significant group differences on goal imitation. We found that there was a non-zero difference in the relation between EMQ-Means End scores and goal imitation between the two groups for EMQ-Means End scores under 11.03 (depicted in light gray in Figure 4). We confirmed this relation by taking a median split of EMQ-Means End scores (score cut off = 11.5) and generating 4 groups dependent on condition and EMQ-Means End scores (1: “Low EMQ-Means End training-first” group; 2: “Low EMQ-Means End imitation-first” group; 3: “High EMQ-Means End training-first” group; 4: “High EMQ-Means End imitation-first” group). Pairwise comparisons between the two Low EMQ-Means End groups indicated a significant difference of goal imitation [*t*_(34)_ = 3.14, *p* =0.002; *W*_*Mann*−*Whitney*_ = 972, *p* < 0.001, corrected for multiple comparisons]. More specifically, among infants who came into the lab with lower EMQ-Means End scores, those in the training-first condition exhibited higher goal imitation (see Figure 3B) than those in the imitation-first condition. This suggests that motor skills did not synergistically boost the effect of training on infants’ action understanding. Rather, training seemed to benefit infants coming in with lower means-end skills, showing a compensatory relation between training and infants’ baseline means-end skills. Second, we examined correlations between EMQ-Means End scores and goal imitation separately in each condition. In the training-first condition, there was no evidence of a relation between EMQ-Means End scores and goal imitation (*r* = −0.26, *p* = 0.121). In the imitation-first condition, EMQ-Means End scores were significantly correlated with goal imitation (*r* = 0.36, *p* = 0.026; *rho* = 0.31, *p* = 0.057; Figure 4). More specifically, infants with better means-end skills were more likely to generate goal responses to the experimenter’s cane-use action.

#### 3.2.1 Replication of prior findings

In conducting this focal analysis, we replicated two findings from the prior literature. We replicated the finding that training alters infants’ understanding of novel goal-directed actions (Sommerville et al., 2005, 2008; Gerson et al., 2015) by demonstrating that infants who experienced cane training prior to the imitation paradigm subsequently imitated the goal of the experimenter’s tool use actions (see Supplementary material for the analyses on the relation between infants’ planful means-ends actions during training and infants’ ability to recognize others’ action as goal-directed). Second, we replicated that infants’ level of means-end skills with an action is associated with recognizing the action as goal-directed (Kanakogi and Itakura, 2011; Cannon and Woodward, 2012; Melzer et al., 2012). That is, when no prior experience was provided beforehand, infants depended on the means-end motor skills that they have accrued through development (imitation-first condition).

#### 3.2.2 Attention during goal imitation

**FIGURE 5:**
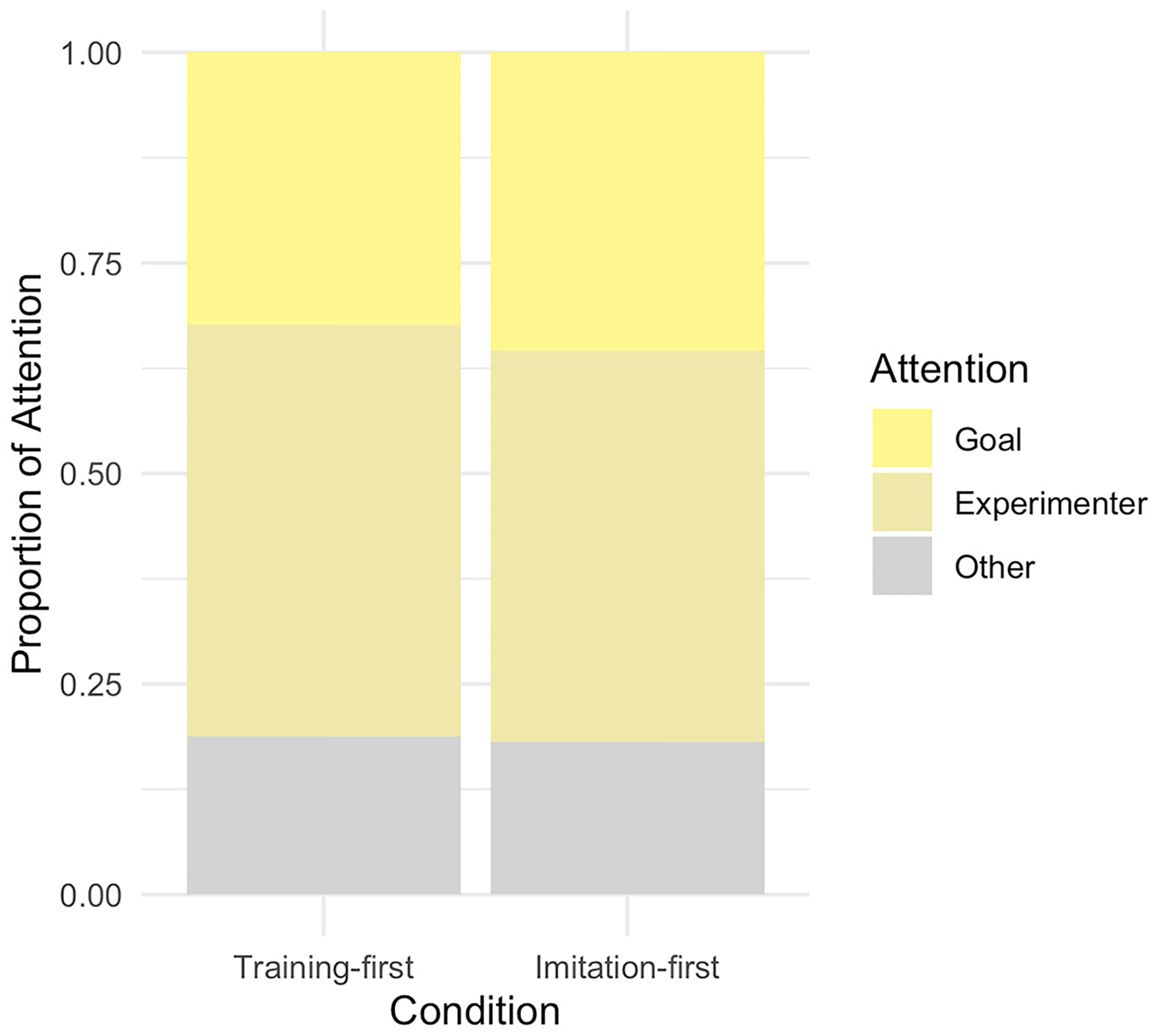
Proportion of visual attention allocation during the goal imitation paradigm.

Follow-up analyses examined whether attentional differences during the imitation session could explain the condition differences on training. There were no condition differences in the proportion of duration of attention the goal, experimenter, or the non-goal toy during goal imitation paradigm (all *p*’s > 0.3; Figure 5); indicating that on average infants in both conditions attended to the scene similarly. Therefore, the training effect on goal imitation was not due to changes in low-level attentional patterns to others’ means-end action.

## 4 Discussion

To identify how action training and an infant’s starting state of means-end skills interact to shape goal understanding, we provided cane training, obtained a metric of infants’ means-end skills using the EMQ, and assessed goal understanding using a goal imitation paradigm. This study provides evidence that action understanding is shaped both by training and infant’s starting state means-end capacity. Training improved action understanding, particularly for those infants who started out with lower means-end skills. Results further indicated that infants who did not receive any training experience in the lab drew on their existing means-end skills. Specifically, those who came into the lab with higher means-end abilities showed better goal understanding. This pattern was specific to means-end skills—no relation was found with gross and fine motor skill.

First, we contribute to the large body of literature on unique effects of training by demonstrating that training on novel tool-use action alters on action understanding of others’ tool-use action (Sommerville et al., 2005, 2008; Skerry et al., 2013; Gerson and Woodward, 2014a,b; Gerson et al., 2015). The infants who received training in a cane-pulling task subsequently generated selective goal responses to the experimenter’s cane-use action. Moreover, we also replicate that infants’ own action capacities relates to action understanding (Sommerville and Woodward, 2005; Kanakogi and Itakura, 2011; Loucks and Sommerville, 2012; Ambrosini et al., 2013). There was a positive association between means-end skills and goal imitation in those who did not receive training beforehand; such that those who came in with higher means-end abilities showed higher goal responses. Importantly, this was not predicted by other metrics of global motor development. This suggests that the relation between infants’ own action experience and action understanding is action specific rather than influenced by general improvements in motor development. However, we also acknowledge that interpreting these main effects should be approached with caution, given the presence of interaction effects in our study.

The current findings go beyond prior work in demonstrating that infants’ means-end skills interact with training experience in their analysis of others’ actions. Our results provide evidence of a compensatory effect of training and motor skills: infants in the training first condition benefitted from the training, and those who came in with less advanced means-end skills who seemed to benefit the most from training. This suggests that training presents differential opportunities that is modulated by infants’ starting-state means-end skills. For infants coming in with less advanced motor skills, training provides an opportunity to use the cane in a coordinated manner and to direct their attention to goals, which they could not yet glean on their own, that supports their understanding of others’ action. However, for those coming in with advanced motor skills, training experience may be priming them on what they already know.

While previous studies have found that infants’ variability in means-ends actions after training was associated with action understanding (Sommerville et al., 2008; Gerson et al., 2015), we did not replicate this association. Interestingly so, even though there was preliminary evidence that those who came in with better means-end skills produced more planful coordinated actions after training, this did not exert a boosting effect on their action understanding in the current study. This suggests that there could be other factors that accounted for a change in infants’ action understanding, beyond information from engaging in goal-directed action. One possibility is that minor differences in our training procedure impacted our ability to detect this effect. In the current study during training, the infant was given the toy at the end of each trial regardless of whether they had successfully pulled the cane. In prior studies (Sommerville et al., 2008; Gerson et al., 2015), the toys were not given to the infants following a non-successful action. This procedural difference may explain why infants in the training condition who were less successful in the training condition still benefitted from training.

Another possibility involves the argument that having multiple instances to acquire a desired toy after attempts of pulling (not dependent on the successfulness of the pull) may have led infants to understand goal of the trained action despite limited proficiency with execution the action itself. Research has shown that highlighting a salient action-effect (even for unfamiliar events such as mechanical claw or back-of-hand toy-touching events) supported action understanding (Jovanovic et al., 2007; Adam and Elsner, 2018; Elsner and Adam, 2021); although see Sommerville et al., 2008; Gerson et al., 2015 for evidence that observational experience with action-effects is not sufficient for action encoding). Lastly, it could be that infants interpreted the training session as a collaborative context—one in which the infant and experimenter act together to retrieve the toy (e.g., if the infant touched the cane and the experimenter gave the toy—as was typical during training). Henderson et al. (2013) have found that 10-month-old infants who engaged in a collaborative activity represented others’ actions in terms of a collaborative goal (see Krogh-Jespersen et al., 2020 for similar results). This may be another explanation for why even the infants who engaged in training but did not necessarily succeed in producing planful actions showed changes in action understanding. Future work should replicate this paradigm without giving the infant the toy during training to determine whether this impacts replication of our effects.

### 4.1 Limitation

The current findings indicate that training improved infants’ sensitivity to the goal structure of others’ actions, particularly for those infants who started out with lower means-end skills. A limitation of our study is the limited variability in means-end skills in our sample, specifically in the training-first condition relative to the imitation-first condition. With a larger sample size encompassing a broader range of means-end skills, we may have explored meaningful effects of means-end skills on understanding others’ actions, also in the training-first condition. Second, the lack of significant improvement between pre- and post-training for the infants in the Imitation-first condition suggests potential differences beyond motor-related abilities. While we suspect this difference is unlikely due to motor-related abilities since there was no significant difference on any dimension of the EMQ, we cannot rule out the possibility that despite random assignment, there could be another unforeseen factor that explains why infants in the imitation-first condition did not learn from training in addition to being fatigue after the imitation paradigm, as well as the observed differences in goal understanding. Third, we restricted infants’ understanding of others’ actions using the same tool and context. Particularly, in the Goal Imitation Task, we used the same cane-use action that the infants had experienced, and we only changed the color of the cane from red to blue. Future studies should address whether means-end skills interact with training in the context of more challenging tasks. For instance, infants entering with high means-end skills may have exhibit a more pronounced synergy effect between training and means-end skills when they encounter an action involving a novel tool. Enhancing both the sample variability of means-end actions, coupled with conducting the goal-understanding paradigm with a novel tool-use action, could yield valuable insights that were not apparent in our current study.

### 4.2 Future directions

Although beyond the scope of this current study, it is an open question regarding the neurocognitive representations that support infants’ analysis of goals. Neuroscientific methods have the advantage over behavioral methods providing information about the processes that underlie perception of actions (Stapel, 2020). Studies investigating the neural correlates during action perception that are associated with motor experience and the correlates associated with action training may shed light on this question. For instance, many studies provide evidence in support of the notion that variation in infants’ motor skills is associated with sensorimotor activity during observation of action that infants have prolonged experience with (van Elk et al., 2008; Cannon et al., 2016; Filippi et al., 2016; Upshaw et al., 2016). Furthermore, recent infant EEG studies showed that visual and motor areas were more connected than other control circuits during observation of familiar grasping action in infants (Debnath et al., 2019; Chung et al., 2022), but not during observation of an unfamiliar cane-use action (Chung et al., 2022). It is also found that infants more competent in grasping objects showed higher levels of motor-visual coupling during action anticipation of both grasping and cane-use action (Colomer et al., 2023). These findings suggest that the neural activity underlying processing and encoding of others’ actions that infants have prolonged experience with may involve an integration of functional connectivity between motor and visual processes, and that this connectivity scales with infants’ motor development. It is an open question whether training of novel action subsequently induces changes in functional connectivity between motor and visual processes or is governed via a different set of networks.

Furthermore, while our work did not find significant differences in overt attention, prior findings indicate that as infants gain proficiency in producing actions themselves, they shift their attention to the goal rather than focusing on the means (Willatts, 1999). The same shift in attention occurs in infants’ perception of others’ means-end actions (Gerson and Woodward, 2013, 2014a). It is possible that training gives rise to differential attention processing that highlights the relation between the agent and the goal, which cannot be accounted by overt attention alone. Neuroscientific methods (e.g., EEG, fNIRS, or fMRI) may provide more sensitive measures of attentional processing (Begus and Bonawitz, 2020; Filippi et al., 2020; Stapel, 2020; Ellis et al., 2021) and may inform whether active training generates changes in attentional processes which in turn modulates action understanding.

## 5 Conclusion

Together this work not only replicates prior findings on the effects of both infants’ own motor skills and direct in-lab intervention experience on infants’ action understanding, but also suggests that the effects of training may differ depending on infants’ motor skills. More specifically, findings highlight a complementary role of infants’ in-lab experience and own motor skills, particularly showing that the effect of training was most beneficial for infants coming in with less means-end skills. This work also demonstrates the value of measuring infant motor development in addition to assessing in-lab trained task performance and provides several avenues for future research examining the relation between active experience and action understanding.

## Supplementary Material

Supplementary Material

## Figures and Tables

**TABLE 1:** Summary of EMQ raw sub-scales scores.

Condition	Visual-reception (VR)	Gross motor (GM)	Fine motor (FM)
	Mean (*SD*)	Mean (*SD*)	Mean (*SD*)
Training-first	2.84 (10.27)	−11.81 (14.07)	−12.16 (12.58)
Imitation-first	1.11 (12.25)	−15.27 (15.27)	−12.67 (12.64)

**TABLE 2:** Selected items for the EMQ-Means End score.

Selected items (#7–13) for the EMQ-Means End scores within the VR scale
**While sitting on you lap or fully supported in a high chair or car seat, you have noticed your child…**
7) shift eye gaze back and forth between your face and an object?
8) focus on a far-away object (e.g., toy across the room?)
9) orient to noises and visually search for the cause of the noise?
10) extend his/her arms toward an object that is close by?
11) pull on a string or cloth to obtain a connected object?
**When your child is sitting on the floor on his/her own without support, your child will…**
12) pull an object to reveal another object that was hidden underneath?
13) find a hidden object when given multiple choices to search?

## Data Availability

The raw data supporting the conclusions of this article will be made available by the authors, without undue reservation.
