# Supplementary material for: Infant action understanding: the roles of active training and motor development
Source: Front Dev Psychol. Author manuscript; Available in PMC 2025 Jan 10. (PMC11720954; doi:10.3389/fdpys.2024.1349031)
Supplement: Supplementary Material [file NIHMS2045198-supplement-Supplementary_Material.pdf]

Supplementary Material

Infant action understanding: The roles of active training and motor development

1 Learning during cane training task, means-end skills and goal imitation

In the training-first condition, seventeen infants performed planful strategies on all three post-training trials, twenty-two infants performed planful strategies on two or fewer post-training trials (2 post-training trials: n=10; 1 post-training trial: n=7; or no post-training trials: n=5) and one infant fussed out during post-training. In the imitation-first condition, twelve infants performed planful strategies on all three post-training trials and twenty-three infants performed planful strategies on two or fewer post-training trials (2 post-training trials: n=17; 1 post-training trial: n=5; or no post-training trials: n=11) and five infants fussed out during post-training.

We took the total number of experimenter prompts provided to each participant to check whether assistance was related to infants' learning. A multivariate regression model with condition and total number of assistance in predicting planful score at post-test(R^2 = 0.32, F(3, 68) = 10.82, p < .001) revealed a main effect of assistance (ß = -0.02, SE=0.01, t=-3.98, p<.001), suggesting that the less amount of experimenter's assistance was associated with infants' post-test planful score, and this was not different between condition.

Next, we evaluated whether we replicated the prior findings of a relation between infants' planful means-ends actions during training and infants' ability to recognize others' action as goal-directed (Sommerville et. al., 2008; Gerson et. al., 2015). In doing so, we also explored whether variations in motor skills affected infants' learning, and thereby, affected changes in infants' action understanding. To answer these questions, we conducted an analysis with the training-first group since these infants demonstrated robust evidence of learning via training (see 3.1.2. Learning during cane training task). First, because pre-test and post-test scores were proportions, we conducted Wilcoxon test with time (pre and post) as a within-subjects factor for each High and Low EMQ-Means End scores by taking a median split of EMQ-Means End scores. Results indicated a significant increase from pre-to-post-test scores in the High Means End scores ($V_{Wilcoxon}$ = 27, *p* = .02), and a marginal increase in the Low Means End scores ($V_{Wilcoxon}$ = 16, *p* = .07), (see Supplemental Figure 1). These findings indicate that training enhanced infants' ability to produce planful actions, and this was stronger for those coming in with higher means-end abilities. Second, we conducted a multivariate regression with difference of pre and post-training scores and EMQ-Means End scores as independent variables and proportion of goal imitation as a dependent variable within the training-first condition. Results revealed no evidence of an effect of the difference in planful scores during training (*p* = .204), effect of EMQ-Means End scores (*p* = .750), nor interaction (*p* = .882) on goal imitation. There was no evidence that the amount of planful strategies that infants learned during the training task nor infants' own means-end skills accounted for infants' subsequent goal imitation in the training-first condition. Thus, we did not replicate the pattern that was found in prior studies.

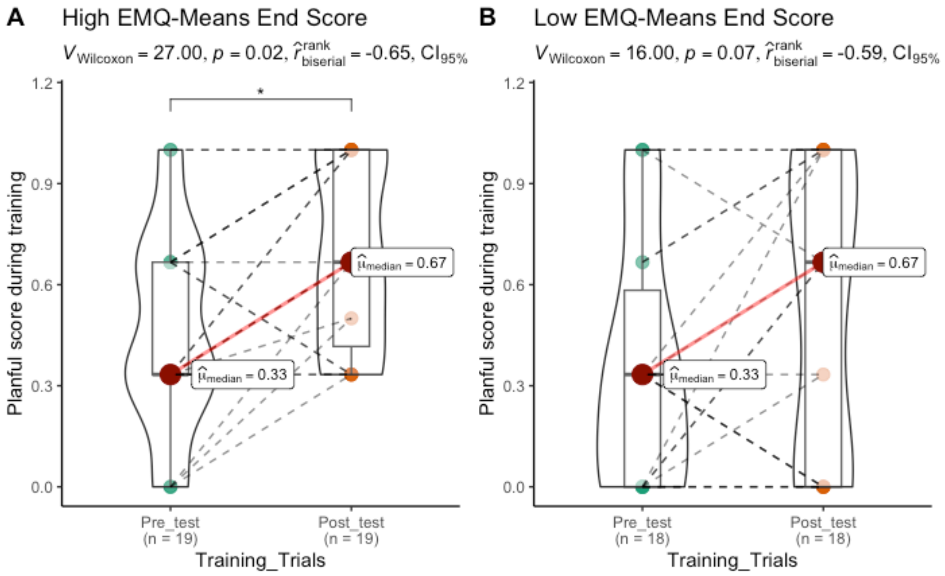

Supplemental Figure 1. Violin plot of infants' Pre-test and Post-test separated by median split of EMQ-Means End scores in the training-first condition.

2 Supplementary Figures and Tables

2.1 Supplementary Figures

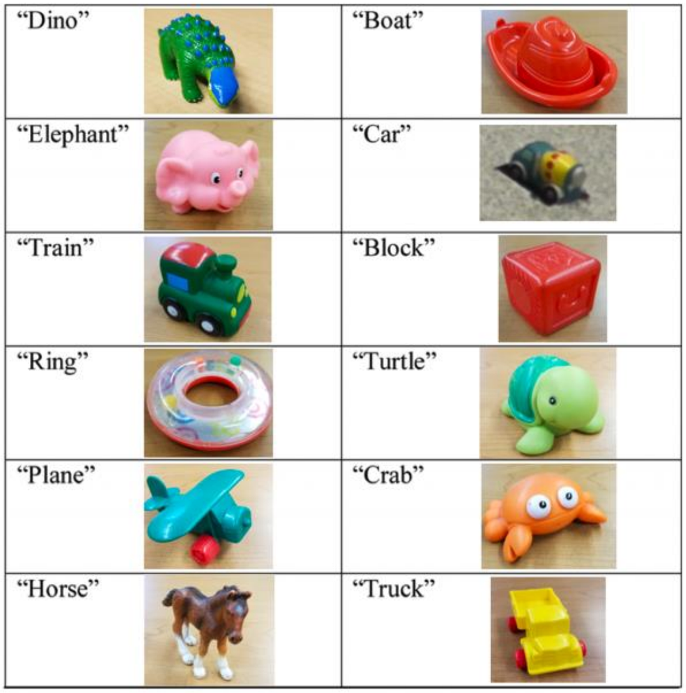

Supplementary Figure 2. Six Pairs of toys that were used in the goal imitation paradigm.

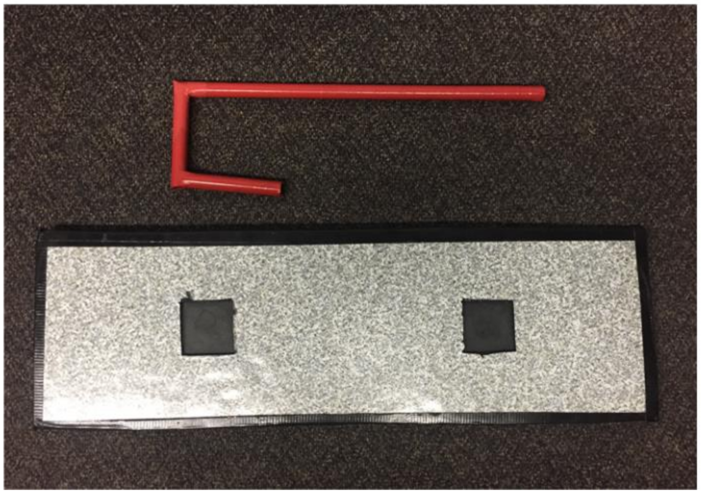

Supplementary Figure 3. Cane and board used in the goal imitation paradigm. (Cane: length of cane: 48cm, width of crook: 13.5cm; Board: 76cm X 23cm)

3 Stepwise regression conducted with all the EMQ subscales

In order to verify that the EMQ items related to means-end problem solving are unique predictors over and other "motor maturity" items in the EMQ, we conducted a stepwise regression (backward selection) starting with all the EMQ subscales (gross motor(GM), fine motor(FM), visual reception items not included in the "means-end" sub-subscale (Non_MeansEnd), and Means End) entered in the model with condition as an interacting factor and goal imitation score as the dependent variable. At each step, the EMQ subscale variable that contributed the least to the model fit, based on a predefined criterion, was removed from the model until we found the best model fit. The predefined criterion was checking for the predictor variable with the smallest t-statistic closest to zero, contributing the weakest evidence to the model. The stopping criteria was to find t-statistic higher than 1. For this analyses, packages 'tidyverse', 'broom', and 'GGally' were used in R.

The first model with all the EMQ subscales entered in indicated that the 'FM' and 'FM and condition' contributed the least to the model fit, so were taken out. The next model with GM, Non_MeansEnd, and Means End scores indicated that the 'Non_MeansEnd' and 'Non_MeansEnd and condition' contributed the least to the model fit, so were taken out. Next, the model with GM and Means End scores indicated that 'GM' and 'GM and condition' contributed least to the model, so were taken out. Finally, our final model with 'Means End' and condition indicated that all the predictor variables contributed to the model, with t-statistic higher than 1(See Supplementary Table 1). The residual plot shows that there are no evident patterns observed (See Supplementary Figure 4). Thus, this final model provides evidence of the unique contribution of the EMQ means-end items and their relations to the goal understanding and training.

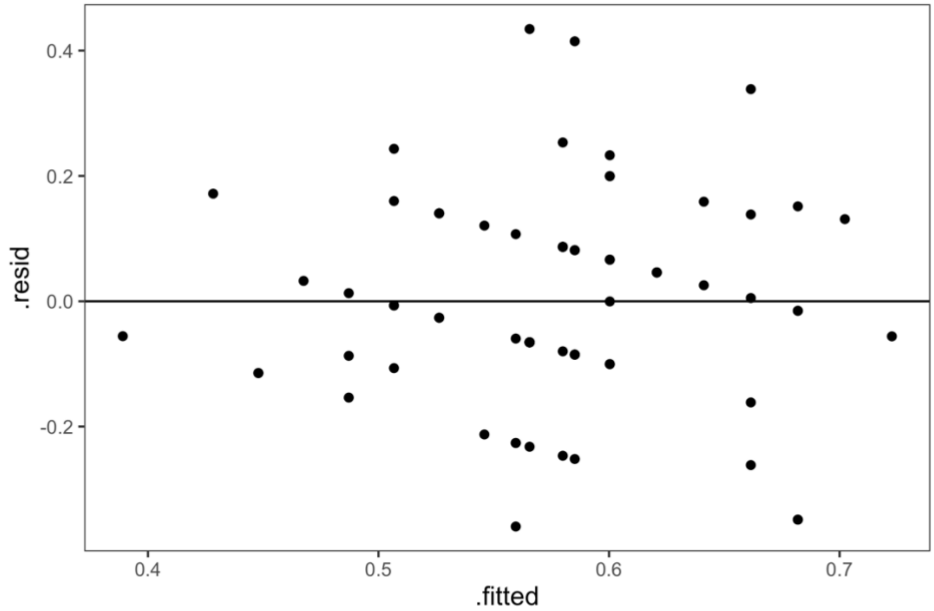

Supplementary Figure 4. Residual plot of the final model from the backward stepwise regression.

Supplementary Table 1. Stepwise Regression Table Summary

	First Model				Second Model				Third Model				Final Model			
Predictors	*Statistic*	*p*	*Estimates*	*standardized std. Error*	*Statistic*	*p*	*Estimates*	*standardized std. Error*	*Statistic*	*p*	*Estimates*	*standardized std. Error*	*Statistic*	*p*	*Estimates*	*standardized std. Error*
(Intercept)	4.18	**<0.01**	0.84	0.17	4.53	**<0.01**	0.84	0.16	5.49	**<0.01**	0.86	0.16	6.19	**<0.01**	0.85	0.15
condition	-2.23	**0.03**	-0.59	0.24	-2.34	**0.02**	-0.58	0.23	-2.74	**0.01**	-0.59	0.23	-2.88	**0.01**	-0.53	0.22
GM	0.32	0.75	0.00	0.20	0.33	0.74	0.00	0.18	0.31	0.76	0.00	0.18				
FM	0.04	0.97	0.00	0.22												
Non MeansEnds	-0.24	0.81	-0.00	0.24	-0.21	0.83	-0.00	0.20								
Means end	-1.27	0.21	-0.02	0.21	-1.35	0.18	-0.02	0.20	-1.61	0.11	-0.02	0.18	-1.68	0.10	-0.02	0.17
condition × GM	-0.48	0.63	-0.00	0.27	-0.56	0.57	-0.00	0.26	-0.51	0.61	-0.00	0.24				
condition × FM	-0.27	0.79	-0.00	0.34												
condition × Non MeansEnds	0.36	0.72	0.00	0.33	0.21	0.83	0.00	0.27								
condition × Means end	2.09	**0.04**	0.04	0.28	2.17	**0.03**	0.04	0.26	2.41	**0.02**	0.04	0.24	2.45	**0.02**	0.04	0.22
Observations	70				71				73				75			
R^2 / R^2 adjusted	0.152 / 0.025				0.150 / 0.056				0.150 / 0.087				0.139 / 0.102			
